# Optimal Design of the Cement, Fly Ash, and Slag Mixture in Ternary Blended Concrete Based on Gene Expression Programming and the Genetic Algorithm

**DOI:** 10.3390/ma12152448

**Published:** 2019-07-31

**Authors:** Xiao-Yong Wang

**Affiliations:** Department of Architectural Engineering, Kangwon National University, Chuncheon 24341, Korea; wxbrave@kangwon.ac.kr; Tel.: +82-33-250-6229

**Keywords:** cost, CO_2_ emissions, fly ash, slag, gene expression programming, genetic algorithm

## Abstract

Concrete producers and construction companies are interested in improving the sustainability of concrete, including reducing its CO_2_ emissions and the costs of materials while maintaining its mechanical properties, workability, and durability. In this study, we propose a simple approach to the optimal design of the fly ash and slag mixture in blended concrete that considers the carbon pricing, material cost, strength, workability, and carbonation durability. Firstly, the carbon pricing and the material cost are calculated based on the concrete mixture and unit prices. The total cost equals the sum of the material cost and the carbon pricing, and is set as the optimization’s objective function. Secondly, 25 various mixtures are used as a database of optimization. The database covered a wide range of strengths between 25 MPa and 55 MPa and a wide range of workability between 5 and 25 cm in slump. Gene expression programming is used to predict the concrete’s strength and slump. The ternary blended concrete’s carbonation depth is calculated using the efficiency factors of fly ash and slag. Thirdly, the genetic algorithm is used to find the optimal mixture under various constraints. We provide examples to illustrate the design of ternary blended concrete with different strength levels and environmental CO_2_ concentrations. The results show that, for a suburban region, carbonation durability is the controlling factor in terms of the design of the mixture when the design strength is less than 40.49 MPa, and the compressive strength is the controlling factor in the design of the mixture when the design strength is greater than 40.49 MPa. For an urban region, the critical strength for distinguishing carbonation durability control and strength control is 45.93 MPa. The total cost, material cost, and carbon pricing increase as the concrete’s strength increases.

## 1. Introduction

Fly ash and slag are industrial byproducts and are widely used as mineral admixtures in the concrete industry. Fly ash and slag have many advantages, such as reducing CO_2_ emissions, lowering material costs, and improving workability and late–age strength. However, the addition of fly ash and slag to concrete reduces its carbonation resistance. To rationally use fly ash and slag to achieve sustainable concrete use, a careful examination of their positive and negative effects should be carried out [1,2].

Many studies have evaluated the sustainability of blended concrete. Celik et al. [3] found that replacing up to 55% of the cement in concrete with a high volume of fly ash and limestone can lower the concrete’s global warming potential. Tae et al. [4] reported that a blend of slag and high–strength concrete can reduce a building’s life–cycle energy and CO_2_ emissions. Rivera et al. [5] showed that 1 m^3^ of concrete containing 728 kg of fly ash has a strength greater than 30 MPa, higher chloride penetration resistance, and a lower environmental impact. Tait and Cheung [6] found that fly ash and slag can be used to produce concrete with no performance loss and a reduced environmental impact. Yang et al. [7] reported that alkali–activated slag foamed concrete has a lower global warming potential and photochemical oxidation potential than Portland cement foamed concrete. Wang et al. [8] showed that when a concrete structure’s durability requirement is met, fly ash can reduce the economic burden and produce cost savings in the long–term.

Compared with studies that evaluate sustainability [3,4,5,6,7,8], there is an insufficient number of studies on the design of a sustainable concrete mixture. Lee et al. [9] proposed a technique, based on the genetic algorithm, neural networks, and a convex hull, for finding the optimal concrete mixture with the lowest cost. Lee and Yoon [10] presented a procedure, based on neural networks and the harmony search algorithm, for designing a concrete mixture under various constraints. Kim et al. [11] proposed an evolution algorithm for minimizing a concrete’s life–cycle cost or CO_2_ emissions that considers the mixing stage, the transportation stage, and the manufacturing stage. Sebaaly et al. [12] proposed a technique, based on a neural network and the genetic algorithm, for optimizing the aggregate gradation and binder content in an asphalt mix. Tapali et al. [13] presented a numerical iteration method for finding the optimal concrete mixture that considers strength, carbonation, and chloride durability. However, the models [9,10,11,12,13] have some weak points. Tapali et al. [13] do not consider the concrete’s workability. Lee et al. [9], Lee and Yoon [10], Kim et al. [11], and Sebaaly et al. [12] do not consider the carbonation durability constraint. Although blended concrete’s strength is the same as that of plain concrete, blended concrete’s carbonation durability is lower than that of plain concrete because pozzolanic reactions occur in the mineral admixtures. Hence, with respect to the design of a blended concrete mixture, strength alone is insufficient to guarantee carbonation durability, such that we should consider carbonation to be a necessary constraint in the design of a blended concrete mixture. Furthermore, environmental CO_2_ concentrations will affect the carbonation rate and have an impact on blended concrete mixtures. For example, the CO_2_ concentration in a suburban region and the CO_2_ concentration in an urban region can be different. Therefore, the strengths and mixtures that are required to produce carbonation durability in the two regions may be different. In summary, with respect to the design of blended concrete mixtures, the strength, carbonation durability, and workability requirements should be met while the material cost and the carbon pricing should be kept as low as possible.

To overcome the limitations of the models [9,10,11,12,13], this study proposes a simple approach to the optimal design of a fly ash and slag blended concrete mixture that considers the carbon pricing, material cost, strength, workability, and carbonation durability. The total cost is set as the optimization’s objective function. Gene expression programing is used to evaluate the concrete’s strength and slump. The Genetic algorithm is used to determine the optimal mixture under various constraints.

## 2. Optimization of the Proportions in the Concrete Mix

To optimize the proportions of fly ash and slag in the blended ternary concrete, the object function and constraint conditions need to be established. In this study, we set the object function as the sum of the material cost and carbon pricing. The constraint conditions include the desired strength, workability, component contents, component ratios, absolute volume, and carbonation durability [14,15].

### 2.1. Object Function

The optimization’s object function is written as follows:

(1) $$\mathrm{COS}T=COST_{M}+COST_{CO2}$$

where COST is the cost of the concrete, $COST_{M}$ is the material cost of the concrete, and $COST_{CO2}$ is the carbon pricing. For a fly ash and slag concrete blend, the concrete’s material cost can be calculated using the concrete component and the component’s unit price as follows:

(2) $${\mathrm{COS}T}_{M}={\mathrm{Pr}}_{C}\times C+{\mathrm{Pr}}_{SG}\times SG+{\mathrm{Pr}}_{FA}\times FA+{\mathrm{Pr}}_{W}\times W+{\mathrm{Pr}}_{CA}\times CA+{\mathrm{Pr}}_{S}\times S+{\mathrm{Pr}}_{SP}\times SP$$

where ${\mathrm{Pr}}_{C}$, ${\mathrm{Pr}}_{SG}$, ${\mathrm{Pr}}_{FA}$, ${\mathrm{Pr}}_{W}$, ${\mathrm{Pr}}_{CA}$, ${\mathrm{Pr}}_{S}$, and ${\mathrm{Pr}}_{SP}$ are the unit prices of the cement, slag, fly ash, water, coarse aggregate, fine aggregate, and superplasticizer, respectively. The concrete component’s unit prices are shown in Table 1. C, SG, FA, W, CA, S, and SP are the masses of the cement, slag, fly ash, water, coarse aggregate, fine aggregate, and superplasticizer in the concrete, respectively. 

The carbon pricing can be calculated based on the CO_2_ emissions content and the unit price of CO_2_ as follows:

(3) $$\begin{array}{l} {\mathrm{COS}T}_{CO2}={\mathrm{Pr}}_{CO2}\times M_{CO_{2}}\\ ={\mathrm{Pr}}_{CO2}\times (CO_{2-C}\times C+CO_{2-SG}\times SG+CO_{2-FA}\times FA+CO_{2-W}\times W+CO_{2-CA}\times CA+CO_{2-S}\times S+CO_{2-SP}\times SP) \end{array}$$

where ${\mathrm{Pr}}_{CO2}$ is the unit price of CO_2_, $M_{CO_{2}}$ is the CO_2_ emissions content, and $CO_{2-C}$, $CO_{2-SG}$, $CO_{2-FA}$, $CO_{2-W}$, $CO_{2-CA}$, $CO_{2-S}$, and $CO_{2-SP}$ are the unit CO_2_ emissions of cement, slag, fly ash, water, coarse aggregate, fine aggregate, and superplasticizer, respectively [7]. The concrete component’s unit CO_2_ emissions are shown in Table 1. We set the value of the CO_2_’s unit price to 0.4817 NT dollar/kg [15]. The contents of CO_2_ emissions are taken from the life–cycle inventory (LCI) of Korea. LCI offers straight–forward accounting of everything involved in the “system” of interest. The data of LCI relates to all the flows in and out of the product system. For example, emissions of CO_2_ from Portland cement production include direct emissions and indirect emissions. Direct CO_2_ emissions mainly come from carbonate decomposition from raw material and the burning of cement kiln fuel. Indirect CO_2_ emissions mainly come from electricity consumption. Considering both direct and indirect CO_2_ emissions, according to the life–cycle inventory (LCI) of Korea, the CO_2_ emission of 1 kg cement is set as 0.93 kg (direct CO_2_ 0.78 kg and indirect CO_2_ 0.15 kg) [7].

### 2.2. Constraint Conditions

The object function (the minimum value of the total cost) is subject to various constraints. Examples are concrete strength, workability, component contents, component ratios, absolute volume, and carbonation durability [14]. Since the addition of fly ash and slag lowers the concrete’s carbonation resistance, carbonation durability must be considered when designing a concrete mixture that contains fly ash and slag [1,2].

The strength constraint is that the designed strength should be higher than the required strength. The formula for the strength constraint is as follows [14]:

(4) real strength≥required strength

Equation (5) shows the workability constraint for fresh concrete [14]:

(5) real slump≥required slump

Equation (6) shows the carbonation durability constraint for a fly ash and slag concrete blend [14]:

(6) carbonation depth≤cover depth.

The range of component contents is as follows:

(7) lower≤component≤upper

where “component” represents the cement, fly ash, slag, binder, water, fine aggregate, coarse aggregate, and superplasticizer. Table 2 shows the concrete component’s lower and upper limits [14].

The component ratio constraint is as follows:

(8) $$R_{l}\leq R_{i}\leq R_{u}$$

where $R_{i}$ is the component ratio (e.g., water–to–binder ratio, water–to–cement ratio, fly–ash–to–binder ratio), and $R_{l}$ and $R_{u}$ are the component ratio’s lower and upper limits, respectively. Table 3 shows details on the component ratio constraint [14]. The sand ratio means the ratio of the mass of fine aggregate to the sum of masses of fine aggregate and coarse aggregate. The mass of binder is equal to the sum of the masses of cement, slag, and fly ash. The ratio of slag to binder means the ratio of the mass of slag to the mass of binder. The ratio of fly ash to binder means the ratio of the mass of fly ash to the mass of binder. The ratio of slag to binder and ratio of fly ash to binder are not constants but depend on specific mixtures of concrete.

The absolute volume constraint is as follows:

(9) $$\frac{W}{\rho_{W}}+\frac{C}{\rho_{C}}+\frac{SG}{\rho_{SG}}+\frac{FA}{\rho_{FA}}+\frac{S}{\rho_{S}}+\frac{CA}{\rho_{CA}}+\frac{SP}{\rho_{SP}}+V_{air}=1$$

where $\rho_{W}$, $\rho_{C}$, $\rho_{SG}$, $\rho_{FA}$, $\rho_{S}$, $\rho_{CA}$, and $\rho_{SP}$ are the densities of the water, cement, slag, fly ash, sand, coarse aggregate, and superplasticizer, respectively, and $V_{air}$ is the volume of the air in the concrete. In this study, the densities of water, cement, slag, fly ash, sand, coarse aggregate, and superplasticizer are 1000 kg/m^3^, 3150 kg/m^3^, 2850 kg/m^3^, 2220 kg/m^3^, 2660 kg/m^3^, 2540 kg/m^3^, and 1220 kg/m^3^, respectively. The specific gravity equals the ratio of the density of a material to the density of water. Equation (9) implies that the sum of each concrete component should equal 1 m^3^ [14].

### 2.3. Evaluation of the Fly Ash and Slag Concrete Blend’s Properties

Yeh [14] conducted experimental studies on a fly ash and slag concrete blend’s strength and slump. The concrete’s 28–day compressive strength ranged from 25 MPa to 55 MPa, and the concrete’s slump ranged from 5 cm to 25 cm. The water–to–binder ratio ranged from 0.3 to 0.7. Table 2 shows the upper and lower limits of the concrete component’s content. Table 3 shows the upper and lower limits of the concrete component’s ratio.

Gene expression programming (GEP) was used to mathematically model strength and slump. GEP is a data–mining technology, proposed by Ferreira [16], based on a gene expression law in biogenetics. GEP combines and develops the Genetic algorithm (GA) and genetic programming (GP). GEP overcomes the shortcomings of GA and GP. The GA’s simple encoding can only deal with simple problems, while GP’s complex encoding leads to code expansion. GEP uses a linear, fixed–length, and tree–like gene coding form. It has a strong function discovery ability and a high search efficiency. GEP’s results are parse trees, which are called expression trees. GEP consists of a function set, a terminal set, a fitness evaluation, control parameters, and a termination condition [16]. Additionally, GEP has some advantages over neural networks [9,10]. A neural network is likely to fall into a local extremum because it is a local search optimization method; GEP is a global optimization method [16].

#### 2.3.1. Strength Model

**Figure 1:**
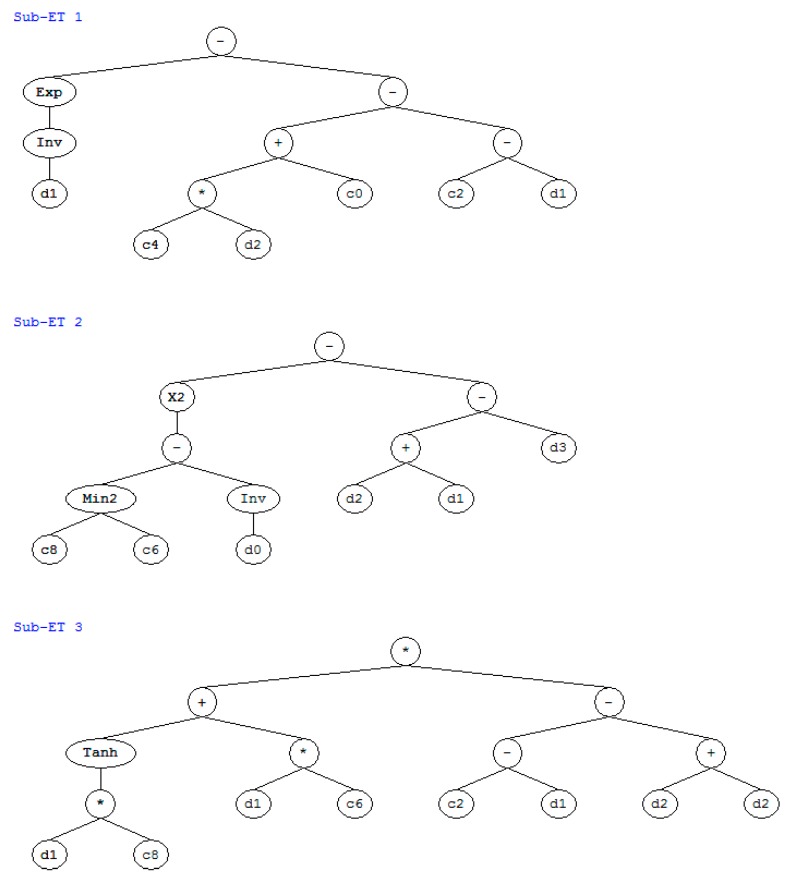
Expression tree for strength.

Based on the experimental results on compressive strength [14] and the GEP method [16], we performed a regression on the concrete’s strength at 28 days as a function of the water–to–binder ratio, the fly–ash–to–binder ratio, the slag–to–binder ratio, and the water content. Figure 1 shows the expression tree for the strength. The strength equals the sum of the three subexpression trees (ET1, ET2, and ET3). The variables d0, d1, d2, and d3 in the subexpression trees denote the water content, water–to–binder ratio, fly–ash–to–binder ratio, and slag–to–binder ratio, respectively. The GEP equation for calculating the strength is as follows:

(10) $$\begin{array}{cc} \mathrm{strength} & =(exp((1.0/(d1)))-(((12.27\times d2)-9.14)-(-12.04-d1)))\\ & +(((-7.212-(1.0/(d0)){)}^{2})-((d2+d1)-d3))\\ & +((tanh((d1\times 3.392))+(d1\times 4.732))\times ((-5.648-d1)-(d2+d2))) \end{array}$$

**Figure 2:**
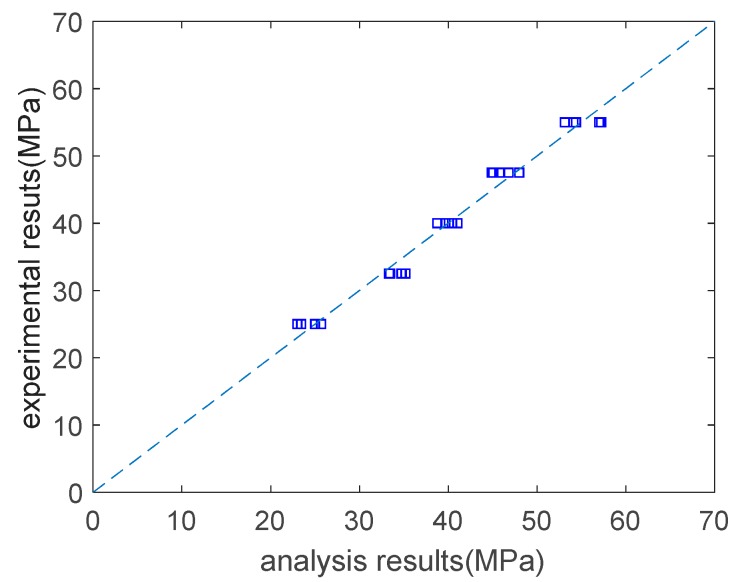
Experimental results vs. analysis results on strength.

**Figure 3:**
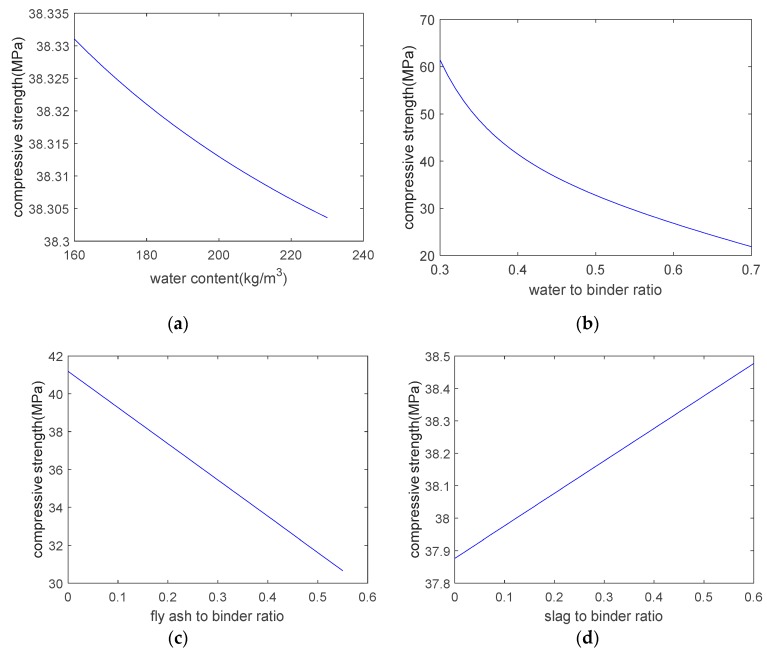
Parameter analysis of strength. (**a**) effect of water content on strength; (**b**) effect of water–to–binder ratio on strength; (**c**) effect of fly–ash–to–binder ratio on strength; (**d**) effect of slag–to–binder ratio on strength.

As shown in Figure 2, the analysis results are in general agreement with the experimental results. The correlation coefficient between the analysis results and the experimental results on the concrete’s strength is 0.99. Based on the expression tree shown in Figure 1, a parameter analysis was carried out that considered the individual effects of the water content, the water–to–binder ratio, the fly–ash–to–binder ratio, and the slag–to–binder ratio on the strength. When conducting the parameter analysis, only one variable at a time was changed, and the other three variables were kept constant. Figure 3 shows the results of the parameter analysis. As the water content and water–to–binder ratio increase, the concrete’s strength decreases (Figure 3a,b). This is due to the increase in the concrete’s porosity. In addition, in Figure 3a, the vertical scale is very closed. This is because the compressive strength is not sensitive to water content. In other words, if we set the water–to–binder ratio, fly–ash–to–binder ratio, and slag–to–binder ratio as constants, and only change water content, the variation in the compressive strength is marginal. As shown in Figure 3a,b, the compressive strength is more sensitive to the water–to–binder ratio than it is to the water content. Moreover, as the fly–ash–to–binder ratio increases, the concrete’s strength decreases (Figure 3c). This is because the fly ash’s reactivity is lower than that of the cement [17]. As the slag–to–binder ratio increases, the concrete’s strength slightly increases (Figure 3d). This is due to the contribution of the slag reaction [17].

#### 2.3.2. Slump Model

**Figure 4:**
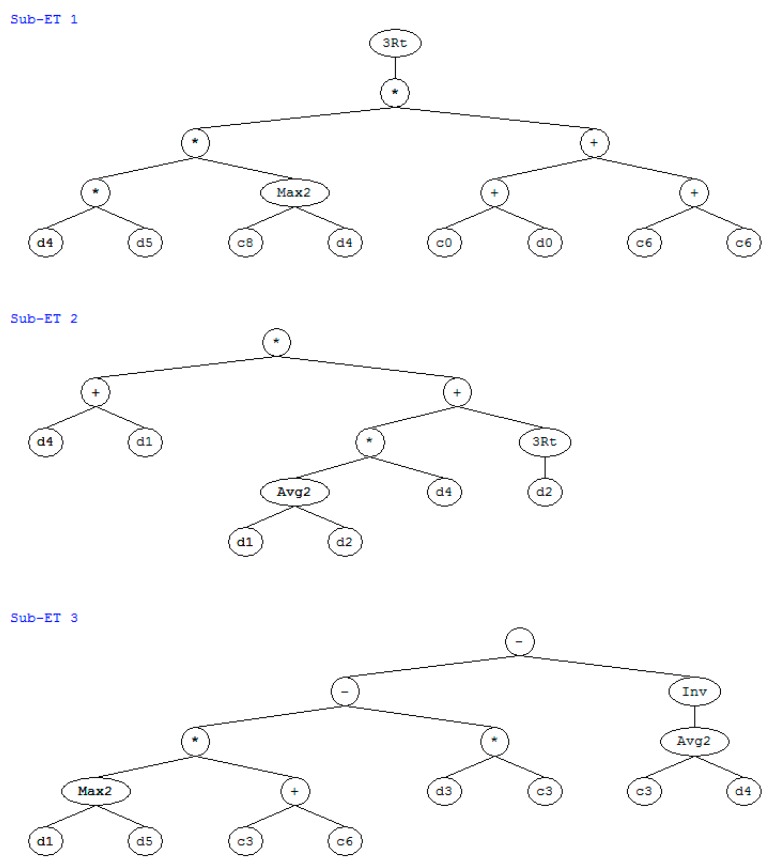
Expression tree for slump.

**Figure 5:**
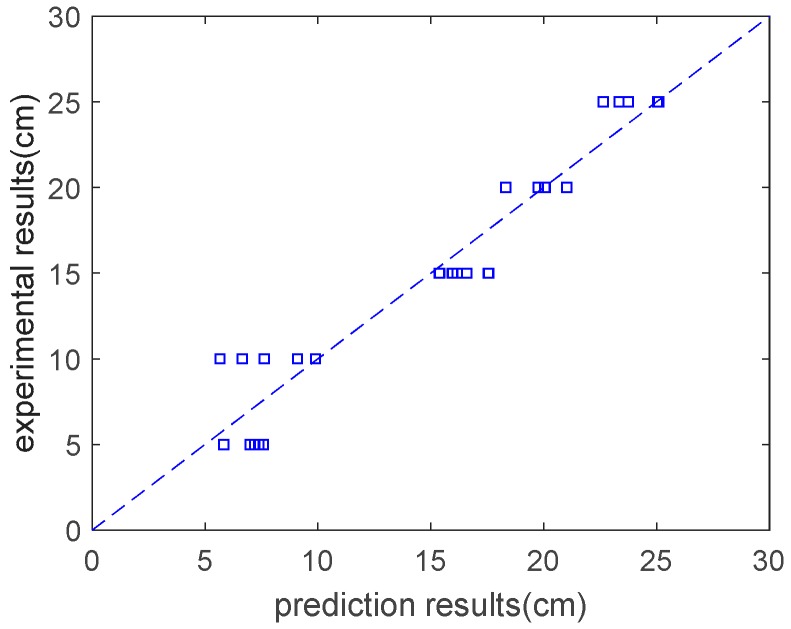
Experimental results vs. prediction results on slump.

**Figure 6:**
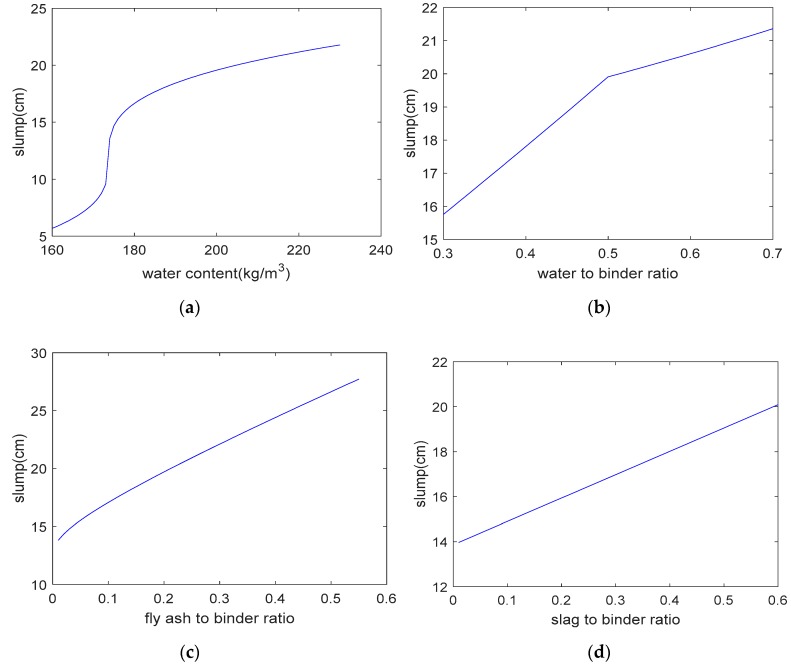

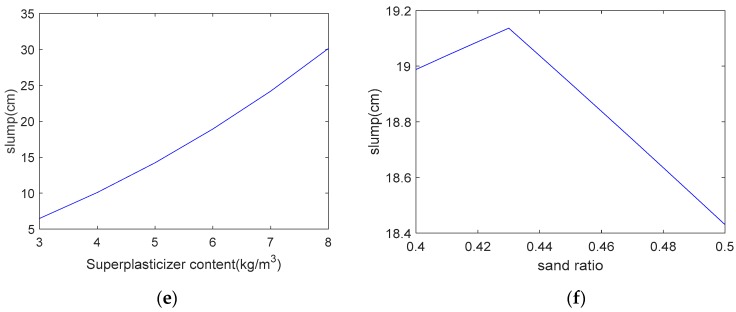
Parameter analysis of slump. (**a**) Effect of water content on slump; (**b**) effect of water–to–binder ratio on slump; (**c**) effect of fly–ash–to–binder ratio on slump; (**d**) effect of slag–to–binder ratio on slump; (**e**) effect of superplasticizer content on slump; (**f**) effect of sand ratio on slump.

Based on the experimental results on slump [14] and the GEP method [16], we performed a regression on the concrete’s slump as a function of the water–to–binder ratio, the fly–ash–to–binder ratio, the slag–to–binder ratio, the water content, the superplasticizer content, and the sand ratio. Figure 4 shows the expression tree for slump. The slump equals the sum of the three subexpression trees (ET1, ET2, and ET3). The variables d0, d1, d2, d3, d4, and d5 in the subexpression trees denote the water content, water–to–binder ratio, fly–ash–to–binder ratio, slag–to–binder ratio, superplasticizer content, and sand ratio, respectively. The GEP equation for calculating slump is as follows:

(11) $$\begin{array}{cc} y= & \mathrm{gep}3\mathrm{Rt}((((d4\times d5)\times \max(-8.800,d4)\times ((1.980+d0)+(\;-175.69))))\\ & +\;((d4+d1)\times ((((d1+d2)/2.0)\times d4)+\mathrm{gep}3\mathrm{Rt}(d2)))\\ & +\;(((\max(d1,d5)\times (-14.674))-(d3\times -10.383))-(1.0/(((-10.383+d4)/2.0)))) \end{array}$$

where gepR3t is a cube root function. As shown in Figure 5, the analysis results are in general agreement with the experimental results. The correlation coefficient between the analysis results and the experimental results regarding the concrete’s slump is 0.96. Based on the expression tree shown in Figure 4, a parameter analysis was carried out that considered the individual effect of the water content, the water–to–binder ratio, the fly–ash–to–binder ratio, the slag–to–binder ratio, the superplasticizer content, and the sand ratio on slump. When we performed the parameter analysis, we only changed one variable at a time, and kept the other four variables constant. Figure 6 shows the results of the parameter analysis for slump. As shown in this figure, as the water content, water–to–binder ratio, fly–ash–to–binder ratio, slag–to–binder ratio, and superplasticizer content increase, the concrete’s slump increases (Figure 6a–e). In addition, the Blaine surface of used fly ash is 323 m^2^/kg. A total of 84% fly ash particles are finer than 45 μm [14]. As the Blaine surface of fly ash is not high, the increase of the fly ash/binder ratio leads to an increase in the slump (shown in Figure 6c). If finer fly ash is used to replace partial cement, the slump of concrete will decrease. On the other hand, as the sand ratio increases, the concrete’s slump first increases and then decreases (Figure 6f). This is due to the fact that low sand content will not provide a sufficient mortar layer for the coarse aggregate and high sand content will increase the total aggregates surface area [14].

#### 2.3.3. Carbonation Model

Papadakis [17,18] proposed a general equation for evaluating the carbonation depth of concrete that contains fly ash and slag. The equation considers both the concrete’s material properties and the environmental exposure conditions, such as relative humidity and temperature. The carbonation depth of a fly ash and slag concrete blend can be determined as follows [17,18]:

(12) $$x_{c}^{}=\sqrt{\frac{2D{\left[CO_{2}\right]}_{0}t}{0.218\times (C+0.7\times SG+0.5\times FA)\times \alpha_{H}}}$$

(13) $$D=6.1\times 10^{-6}{\left(\frac{\left[W-0.267\times (C+0.7\times SG+0.5\times FA)\times \alpha_{H}\right]/1000}{\frac{C+0.7\times SG+0.5\times FA}{\rho_{c}}+\frac{W}{\rho_{w}}}\right)}^{3}{\left(1-\frac{RH}{100}\right)}^{2.2}$$

where $x_{c}^{}$ is the concrete’s carbonation depth, D is the CO_2_ diffusivity, ${\left[CO_{2}\right]}_{0}$ is the CO_2_ molar concentration at the concrete’s surface, $\alpha_{H}$ is the degree of reaction of the binders ($\alpha_{H}=1-\exp(-3.38\times W/(C+0.7\times SG+0.5\times FA))$ [19], and RH is the environmental relative humidity. The carbonation efficiency factors of slag and fly ash are 0.7 and 0.5, respectively [17]. In the denominator of Equation (12), $0.218\times (C+0.7\times SG+0.5\times FA)\times \alpha_{H}$ is the content of carbonatable substances in the concrete. In the numerator of Equation (13), $\left[W-0.267\times (C+0.7\times SG+0.5\times FA)\times \alpha_{H}\right]/1000$ is the carbonated concrete’s porosity.

The effect of environmental temperature on CO_2_ diffusivity can be viewed as complying with the Arrhenius law, as follows [17]:

(14) $$D(T)=D_{ref}\exp\left[\beta (\frac{1}{T_{ref}}-\frac{1}{T})\right]$$

where $D_{ref}$ is the CO_2_ diffusivity at the reference temperature T_ref_ (20 °C), D(T) is the CO_2_ diffusivity at temperature T, and β is the activation energy of CO_2_ (β = 4300).

### 2.4. Summary of the Approach to Design Optimization

In this section, we determine the object function of and the constraints on the concrete mixture’s proportions. The object function is the concrete’s total cost, which is equal to the sum of the material cost and the carbon pricing. The constraints include various types of mechanical and constructability performance. These constraints are shown in Table 4. The GEP method is used to predict the concrete’s compressive strength and slump. The efficiency factors of slag and fly ash are used to determine the blended concrete’s carbonation depth. Concrete mixtures that meet various performance requirements can be acquired once the object function and constraints have been solved.

The genetic algorithm is used to solve the object function under constraints [20,21]. The GA originated from a computer simulation of biological systems. It is a random global search and optimization method that was developed to imitate an evolutionary mechanism of natural organisms. It is an efficient, parallel, and global search method that can automatically acquire knowledge about the search space during the search process and self–adaptively control the search process to obtain the best solution.

The basic steps of the genetic algorithm are: Step 1, generate a random population; Step 2, determine the fitness of the individual and make a selection according to the fitness; Step 3, generate new individuals based on a crossover and mutation operation; and Step 4, check whether the termination criteria are satisfied; if the termination criteria are not satisfied, return to Step 2.

We used the MATLAB global optimization toolbox to solve the objective function under constraints [20,21]. The equation for the object function and constraints can be set in the MATLAB global optimization toolbox. The genetic algorithm can then be used to find the optimal mixture; i.e., the mixture with the minimum cost and that satisfies the given constraint conditions.

## 3. Illustrative Examples and Discussion

In this section, we provide examples to illustrate the design of a fly ash and slag concrete blend mixture with various strength levels (30 MPa, 40 MPa, and 50 MPa). The strengths of these concrete mixes are used to show the necessity of constraining the carbonation durability in the mixture design. The required slump is assumed to be 70 mm, the exposure temperature is assumed to be 20 °C, the carbon dioxide concentration is assumed to be 0.041% (for a suburban region), and the relative humidity is assumed to be 0.65. The exposure temperature and relative humidity correspond to a temperate exposure condition [22,23]. We aim for a 50–year service life. The cover depth is assumed to be 25 mm, which corresponds to the minimum cover depth under a temperate exposure condition [23,24,25]. The air content V_air_ in the concrete mixture is assumed to be 2%.

Particle size distributions of fine aggregate and coarse aggregate are shown in Table 5 [14]. The Blaine surfaces of cement, fly ash, and slag are 334 m^2^/kg, 323 m^2^/kg, and 601 m^2^/kg, respectively. Cement is American Society for Testing and Materials (ASTM) type I cement. Cement consists of lime (63.6%), silica (20.2%), alumina (4.3%), ferrite (3.08%), and a small number of other oxides. Fly ash is a by–product of a fossil power plant. Fly ash consists of silica (49.7%), alumina (27.8%), lime (6.3%), ferrite (5.2%), and a small number of other oxides. Slag is a by–product of the iron and steel industry. Slag consists of lime (41.4%), silica (31.6%), alumina (13.8%), magnesium (7.5%), and a small number of other oxides.

### 3.1. Proportion Design without Considering Carbonation

In this section, we design a fly ash and slag blended concrete without considering carbonation. Based on the Genetic algorithm and under various constraints, such as strength, slump, the concrete component’s range, the component ratio’s range, and absolute volume, the optimal mixtures for different strength levels were determined and are shown in Table 6. Mix1, Mix2, and Mix3 are mixtures that correspond to a strength of 30 MPa, 40 MPa, and 50 MPa, respectively. The concrete component’s value (shown in Table 6) falls within the lower and upper limits (shown in Table 2). The fly ash contents for each mixture are the same; i.e., 200 kg/m^3^, which is equal to the fly ash’s upper limit. This is due to the fact that fly ash’s price and CO_2_ emissions are much lower than those of cement and slag. In addition, in Table 6, Mix1 with 30 MPa and Mix2 with 40 MPa both have the same cement and water content, but the slag content of Mix2 is much higher than that of Mix1. The binder in concrete consists of cement, fly ash, and slag. As Mix2 has a higher binder content, the water–to–binder ratio of Mix2 is lower than that of Mix1. Hence the strength of Mix2 (40 MPa) is higher than that of Mix1 (30 MPa). Moreover, although Mix1 has higher fine and coarse aggregate contents, the slag content of Mix1 is lower than that of Mix2. From Mix1 to Mix2, the increase in the slag volume can compensate for the reductions in the aggregate volume. Hence, the absolute volumes of each mix are the same, i.e., 1 m^3^.

Table 7 shows each mixture’s performance in terms of strength, slump, carbonation depth, carbon pricing, material cost, and total cost. The slump of concrete is determined by using Equation (11). Each mixture’s slump is higher than the required slump. The strength values of Mixes 1, 2, and 3 are determined by using Equation (10) and are reported to two decimal points. As the concrete’s strength increases, its carbonation depth decreases, and its total cost increases.

Table 8 shows the values of the concrete component’s ratios. Firstly, the value of the water–to–solid ratio for each mixture is the same, i.e., 0.08, which is equal to the water–to–solid ratio’s lower limit. Secondly, the value of the superplasticizer–to–binder ratio for each mixture is the same, i.e., 0.013, which is equal to the superplasticizer–to–binder ratio’s lower limit. This is due to the fact that the superplasticizer’s price is much higher than that of the other components. Thirdly, the value of the sand ratio for each mixture is the same, i.e., 0.4, which is equal to the sand ratio’s lower limit. This is due to the fact that the sand’s price is higher than that of the coarse aggregate. Fourthly, as the concrete’s strength increases, the water–to–binder ratio decreases. This trend corresponds to Abram’s law. A lower water–to–binder ratio helps to produce high–strength concrete. Finally, the values of the component ratios fall within the lower and upper limits (shown in Table 3). The sum of each component’s volume is equal to unity (Equation (9)).

**Figure 7:**
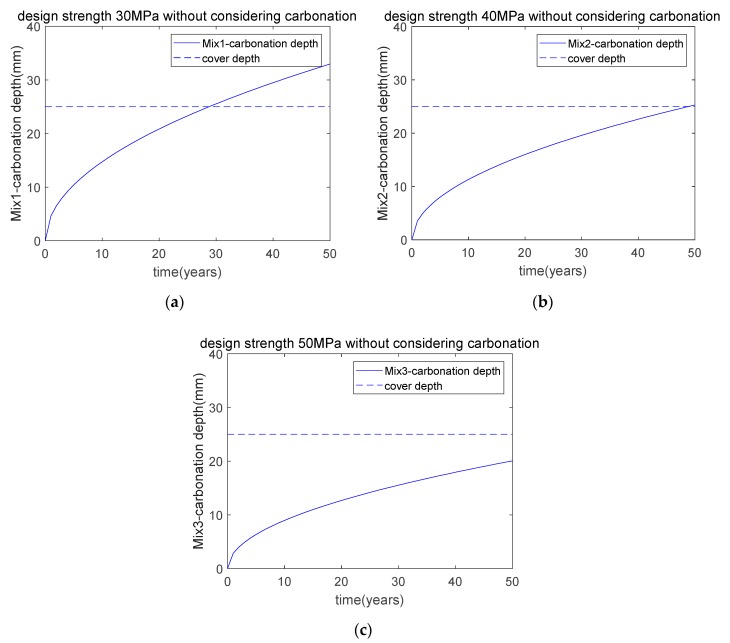
Carbonation depth of concrete without considering carbonation. (**a**) carbonation depth of Mix1; (**b**) carbonation depth of Mix2; (**c**) carbonation depth of Mix3.

We calculated the carbonation depths of Mix1, Mix2, and Mix3 using the carbonation model. Figure 7 shows the carbonation depths of Mix1, Mix2, and Mix3. As shown in Figure 7a, for the concrete with a strength of 30 MPa (Mix1), after 50 years of exposure, the carbonation depth will exceed the cover depth. This is because the addition of fly ash and slag will increase the concrete’s carbonation depth. Similarly, as shown in Figure 7b, the carbonation depth of Mix2 (40 MPa) is also slightly higher than the cover depth. However, as shown in Figure 7c, for the concrete with the highest strength (50 MPa, Mix3), after 50 years of exposure, the carbonation depth will be lower than the cover depth. This means that, as the binder content increases, the concrete’s carbonation resistance also increases, and the carbonation depth will decrease.

Hence, when designing a fly ash and slag concrete blend with a moderate strength, such as 30 MPa or 40 MPa, the carbonation durability requirement cannot be met over the entire service life. When designing a fly ash and slag concrete blend with high strength, such as 50 MPa, the carbonation durability requirement can be met.

### 3.2. Proportion Design in a Suburban Region Considering Carbonation

In this section, we design a fly ash and slag concrete blend mixture while taking carbonation into consideration. We aim at a strength of 30 MPa. In Section 3.1, the carbonation constraint was not considered. In this section, we take the carbonation constraint into consideration.

Based on the genetic algorithm and under various constraints, the concrete mixture was calculated and is shown in Table 6. Mix4 corresponds to a designed strength of 30 MPa, considering the carbonation durability constraint. Table 7 shows the performance of Mix4. Table 8 shows the values of the component ratios.

**Figure 8:**
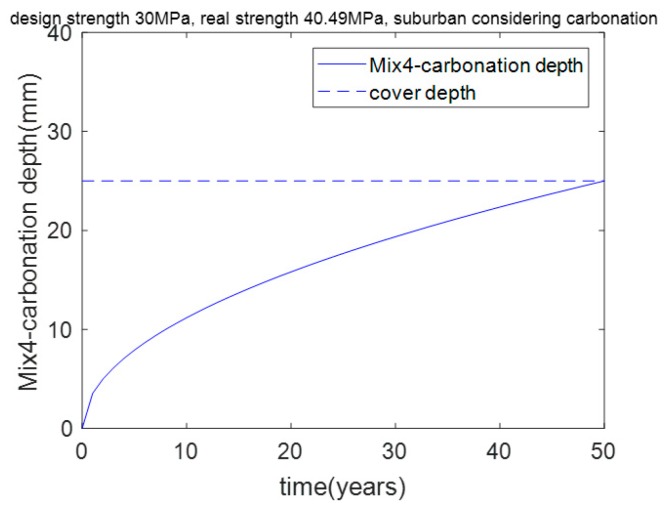
Carbonation depth of Mix4 concrete in a suburban region considering carbonation.

As shown in Table 7, Mix4’s actual strength is 40.49 MPa, which is much higher than the aimed–for strength of 30 MPa. This result means that concrete with a strength of 30 MPa cannot meet the carbonation durability requirement, and concrete with a strength of 40.49 MPa can meet the carbonation durability requirement. In other words, when the aimed–for compressive strength is less than 40.49 MPa, carbonation durability is the dominant factor in the mixture’s design. Furthermore, when the aimed–for compressive strength is greater than 40.49 MPa, strength is the dominant factor in the mixture’s design. As shown in Figure 8, after 50 years of exposure, Mix4’s carbonation depth is equal to the cover depth (25 mm). This means that carbonation is the controlling factor for Mix4.

### 3.3. Proportion Design in an Urban Region Considering Carbonation

In Section 3.2, the CO_2_ concentration was assumed to be 0.041%, which corresponds to the CO_2_ concentration in a suburban region. In an urban region, due to the large amount of CO_2_ emissions from transportation, industrial factories, and residential activities, the CO_2_ concentration is much higher than that in a suburban region. In this section, we consider the effect of a high environmental CO_2_ concentration on the design of a ternary blended concrete mixture. The CO_2_ concentration in an urban region is assumed to be 0.053% (30% higher than that in a suburban region) [25]. The aimed–for strength is assumed to be 30 MPa.

**Figure 9:**
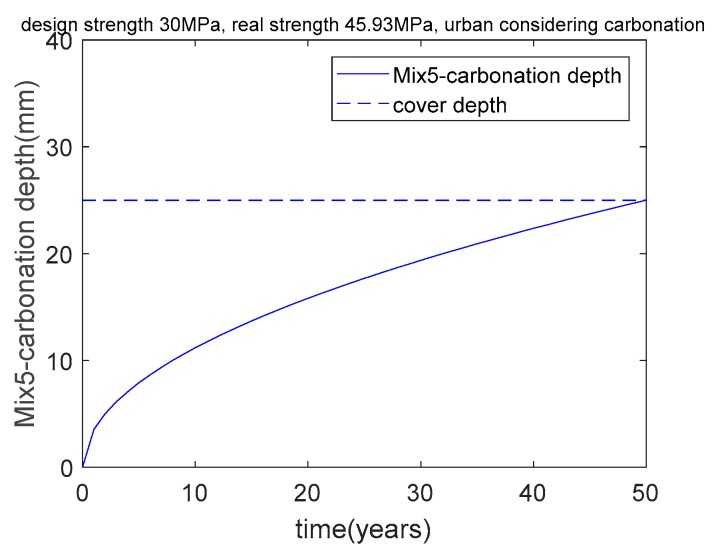
Carbonation depth of Mix5 concrete in an urban region considering carbonation.

**Figure 10:**
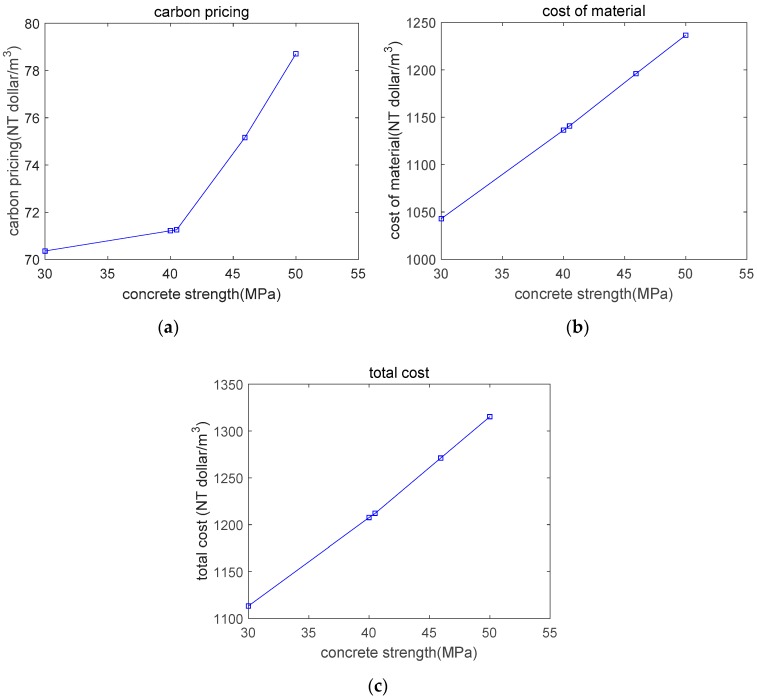
The cost of the concrete mixture. (**a**) carbon pricing; (**b**) cost of concrete material; (**c**) total cost of each mixture.

Based on the genetic algorithm and under various constraints, the concrete mixture was calculated and is denoted as Mix5. As shown in Table 7, Mix5’s real strength is 45.93 MPa, which is much higher than the aimed–for strength of 30 MPa. In other words, for an urban region, 45.93 MPa is the critical strength for distinguishing carbonation durability control and strength control. As shown in Figure 9, after 50 years of exposure, Mix5’s carbonation depth is equal to the cover depth (25 mm). In addition, from a suburban region to an urban region, the CO_2_ concentration increases from 0.041% to 0.053%, and the critical strength for distinguishing carbonation durability control and strength control increases from 40.49 MPa to 45.93 MPa. As shown in Figure 10, as the concrete’s strength increases, the carbon pricing, material cost, and total cost increase.

In this study, we consider the constraints of both strength and carbonation durability. The time of exposure relates to the constraint of carbonation durability. This study assumed that the service life is 50 years. We find that for a suburban region (low CO_2_ concentration region) and an urban region (high CO_2_ concentration region), the critical strengths to meet the requirement of carbonation durability are 40.49 MPa and 45.93 MPa, respectively. An increase in the service life means a strengthening of the constraint of carbonation durability, which is akin to changing exposure conditions from a low CO_2_ concentration region to a high CO_2_ concentration region. Hence, if the aimed service life is higher than 50 years, the critical strengths to meet the requirement of carbonation durability will be higher. In other words, to meet the requirement of a longer service life, the binder content of the optimal critical mixture should be increased.

### 3.4. Discussion

From Section 3.1, Section 3.2 and Section 3.3 we proposed an integrated gene expression programming (GEP)-genetic algorithm (GA) procedure for determining the optimal mixture design of blended concrete. We used GEP for modeling and GA for optimization. We designed a total of five optimal mixtures as the object dataset. To find the general trends of the optimal dataset, we made six additional design cases. The design strengths of additional cases are 32.5, 35, 37.5, 42.5, 45, and 47.5 MPa, respectively. We find that after adding the new design cases, the fundamental trends of optimal results do not change. As the concrete’s strength increases, the carbon pricing, material cost, and total cost increase.

In addition, based on the integrated GEP–GA procedure, we designed a ternary blended concrete using another database. The new database is mainly taken from Yeh’s (1998) study [26]. The new database consists of 425 mixtures with various strengths, whose size is much bigger than the database that was used in Section 3.1, Section 3.2 and Section 3.3. Based on the integrated GEP–GA procedure, we can find the differences and similarities of the optimal mixtures as determined from various databases.

Differences in optimal mixtures: (1) The regression equations of strength from various databases are different. (2) For strength control cases (Mix1, Mix2, and Mix3), the optimal mixtures from various databases are different. This is because the strength equation for various databases are different. (3) The critical strengths for differentiating carbonation control and strength control from various databases are different.

Similarities in the optimal mixtures are as follows: (1) For carbonation durability control cases (Mix4 and Mix5), the optimal mixtures from various databases are the same. This is because the calculation equation of the carbonation depth is the same for various databases. (2) For low–strength blended concrete, carbonation is a control factor of optimal mixture design. While for high–strength blended concrete, strength is a control factor of optimal mixture design. For various databases, this trend is the same. (3) As the strength of the concrete increases, carbon pricing, cost of concrete material, and the total cost of concrete increase. For various databases, this trend is the same.

The method proposed in this study can be regarded as a general method for the design of low–cost and low–CO_2_ blended concrete. To use the proposed method, firstly, we collected the available mixtures and performed regressions on the strength and workability of the concrete using GEP. Secondly, we selected the object function and performed an optimization using GA. For different countries, the strength and slump equations may be different from the equations that were used in this study. Although these equations may differ, the design procedure will remain very similar.

## 4. Conclusions

In this study, we proposed a simple approach to the optimization of the CO_2_ emissions and the material cost of a fly ash and slag concrete blend considering constraints with respect to workability, mechanical properties, and carbonation durability.

Firstly, we calculated the material cost based on the concrete mixture and the component’s unit price. We calculated the carbon pricing based on the levels of CO_2_ emissions and the CO_2_ unit price. The total cost is equal to the sum of the material cost and the carbon pricing and was set as the optimization’s object function.

Secondly, gene expression programming was used to predict the concrete’s strength and slump. The GEP results were found to be in general agreement with the experimental results. With regard to the concrete’s strength and slump, the correlation coefficients between the calculation results and the experimental results were found to be 0.99 and 0.96, respectively. The ternary blended concrete’s carbonation depth was calculated using the efficiency factors of fly ash and slag. Furthermore, the genetic algorithm was used to find the optimal mixture, considering the object function and various constraints.

Thirdly, we provided examples to illustrate the design of ternary blended concrete with different strength levels. The influence of carbonation on the optimal mixture was clarified. For a suburban region, the critical strength for distinguishing carbonation durability control and strength control was found to be 40.49 MPa. When the designed strength is less than 40.49 MPa, carbonation durability is the controlling factor in the mixture’s design. When the designed strength is greater than 40.49 MPa, strength is the controlling factor in the mixture’s design. Given that, in an urban region, the CO_2_ concentration is higher than that in a suburban region, the critical strength was found to be 45.93 MPa, which is higher than that for a suburban region. As the concrete’s strength increases, the carbon pricing, the concrete material cost, and the total cost increase.

In conclusion, the proposed approach can be used as a general tool for designing sustainable concrete. For different countries, the strength and slump equations may differ from the equations that were used in this study; however, the design procedure will be very similar.

## Figures and Tables

**Table 1:** The unit prices and CO_2_ emissions of the concrete component [7,14].

	Unit Prices (NT dollar/kg)	Unit CO_2_ Emissions (kg/kg)
Cement	2.25	0.931
Fly ash	0.6	0.0196
Slag	1.2	0.0265
Water	0.01	0.000196
Superplasticizer	25.1	0.25
Coarse aggregate	0.236	0.0075
Fine aggregate	0.28	0.0026

**Table 2:** The lower and upper limits of the concrete component [14].

	Cement (kg/m^3^)	Fly Ash (kg/m^3^)	Slag (kg/m^3^)	Water (kg/m^3^)	Superplasticizer (kg/m^3^)	Coarse Aggregate (kg/m^3^)	Fine Aggregate (kg/m^3^)
**Lower Limit**	140	0	0	150	3	780	640
**Upper Limit**	350	200	240	250	15	1050	900

**Table 3:** The component ratio constraint [14].

	Water–to–Cement Ratio	Water–to–Binder Ratio	Water–to–Solid Ratio	Superplasticizer–to–Binder Ratio	Fly–Ash–to–Binder Ratio	Slag–to–Binder Ratio	Mineral–Mixtures–to–Binder Ratio	Aggregate–to–Binder Ratio	**Sand Ratio**
**Lower Limit**	0.6	0.3	0.08	0.013	0	0	0.25	2.7	0.40
**Upper Limit**	1.6	0.7	0.12	0.04	0.55	0.6	0.7	6.4	0.52

**Table 4:** Constraints of the mixture design.

Constraints	Equations
Strength constraint	real strength≥required strength
Carbonation durability constraint	carbonation depth≤cover depth
Workability constraint	real slump≥required slump
Component contents constraint	lower≤component≤upper
Component ratios constraint	$R_{l}\leq R_{i}\leq R_{u}$
Absolute volume constraint	$\frac{W}{\rho_{W}}+\frac{C}{\rho_{C}}+\frac{SG}{\rho_{SG}}+\frac{FA}{\rho_{FA}}+\frac{S}{\rho_{S}}+\frac{CA}{\rho_{CA}}+\frac{SP}{\rho_{SP}}+V_{air}=1$

**Table 5:** Particle size distributions of aggregate [14].

Coarse Aggregate		Fine Aggregate	
Sieve Size (mm)	Cumulative Percent Retained (%)	Sieve Size (mm)	Cumulative Percent Retained (%)
20	0	10	0
14	20.7	5	2
10	56.5	2.5	11
5	91.6	1.25	30
2.5	97.5	0.63	57
		0.315	82
		0.160	95

**Table 6:** The mixtures of concrete.

	Cement (kg/m^3^)	Fly Ash (kg/m^3^)	Slag (kg/m^3^)	Water (kg/m^3^)	Superplasticizer (kg/m^3^)	Coarse Aggregate (kg/m^3^)	Fine Aggregate (kg/m^3^)
**Mix1** **(30 MPa)**	140.00	200.00	40.94	167.04	4.95	1024.22	682.82
**Mix2** **(40 MPa)**	140.00	200.00	113.56	167.35	5.90	982.98	655.32
**Mix3** **(50 MPa)**	155.48	200.00	162.78	167.74	6.74	938.48	640.01
**Mix4** **(40.49 MPa–Suburban Considering Carbonation)**	140.00	200.00	116.98	167.36	5.94	981.03	654.02
**Mix5** **(45.93 MPa–Urban Considering Carbonation)**	147.97	200.00	145.27	167.56	6.41	960.78	640.52

**Table 7:** The performance of the concrete.

	Strength (MPa)	Slump (cm)	Carbonation Depth (mm)	Carbon Pricing (NT dollar/m^3^)	Material Cost (NT dollar/m^3^)	Total Cost (NT dollar/m^3^)
**Mix1** **(30 MPa)**	30.00	8.24	32.96	70.36	1043.00	1113.36
**Mix2** **(40 MPa)**	40.00	12.46	25.29	71.22	1136.41	1207.63
**Mix3** **(50 MPa)**	50.00	15.10	20.07	78.71	1236.62	1315.33
**Mix4** **(40.49 MPa–Suburban Considering Carbonation)**	40.49	12.62	25.00	71.26	1140.81	1212.07
**Mix5** **(45.93 MPa–Urban Considering Carbonation)**	45.93	14.17	25.00	75.16	1195.97	1271.13

**Table 8:** The component ratio of each mixture of concrete.

	Water–to–Cement Ratio	Water–to–Binder Ratio	Water–to–Solid Ratio	Superplasticizer–to–Binder Ratio	Fly–Ash–to–Binder Ratio	Slag–to–Binder Ratio	Mineral–Mixtures–to–Binder Ratio	Aggregate–to–Binder Ratio	Sand Ratio	Total Volume (Including Air)
**Mix1**	1.193	0.438	0.080	0.013	0.525	0.107	0.632	4.481	0.400	1.00
**Mix2**	1.195	0.369	0.080	0.013	0.441	0.250	0.691	3.612	0.400	1.00
**Mix3**	1.079	0.324	0.080	0.013	0.386	0.314	0.700	3.046	0.405	1.00
**Mix4**	1.195	0.366	0.080	0.013	0.438	0.256	0.694	3.578	0.400	1.00
**Mix5**	1.132	0.340	0.080	0.013	0.405	0.295	0.700	3.246	0.400	1.00
